# Integrative Analysis of lncRNA-mRNA Profile Reveals Potential Predictors for SAPHO Syndrome

**DOI:** 10.3389/fgene.2021.684520

**Published:** 2021-06-21

**Authors:** Yuxiu Sun, Chen Li, Qingyi Lu, Haixu Jiang, Mengmeng Zhu, Guangrui Huang, Ting Wang

**Affiliations:** ^1^Beijing Research Institute of Chinese Medicine, Beijing University of Chinese Medicine, Beijing, China; ^2^Department of Traditional Chinese Medicine, Peking Union Medical College Hospital, Peking Union Medical College and Chinese Academy of Medical Sciences, Beijing, China; ^3^School of Life Sciences, Beijing University of Chinese Medicine, Beijing, China

**Keywords:** SAPHO syndrome, RNA-seq, lncRNA-mRNA interaction, neutrophil, predictor

## Abstract

Synovitis, acne, pustulosis, hyperostosis, and osteitis (SAPHO) syndrome is known as a rare disease characterized by inflammatory lesions on bones and skin. Polymorphism of clinical manifestation and lack of molecular biomarkers have both limited its diagnosis. Our study performed RNA sequencing (RNA-seq) and integrative bioinformatics analysis of long noncoding RNA (lncRNA)-messenger RNA (mRNA) profile in patients with SAPHO syndrome and healthy controls. A total of 4,419 differentially expressed (DE) mRNAs and 2,713 lncRNAs were identified (*p* < 0.05, fold change > 2) and a coexpression network was constructed to further investigate their regulatory interactions. The DE lncRNAs were predicted to interact with mRNAs in both *cis* and *trans* manners. Functional prediction found that the lncRNA-targeted genes may function in SAPHO syndrome by participating in biological process such as adipocytokine signaling pathway, ErbB signaling pathway, FoxO signaling pathway, as well as production and function of miRNAs. The expression levels of three pairs of coexpressed lncRNA-mRNAs were validated by qRT-PCR, and their relative expression levels were consistent with the RNA-seq data. The deregulated RNAs GAS7 and lnc-CLLU1.1-1:2 may serve as potential diagnostic biomarkers, and the combined receiver operating characteristic (ROC) curve of the two showed more reliable diagnostic ability with an AUC value of 0.871 in distinguishing SAPHO patients from healthy controls. In conclusion, this study provides a first insight into long noncoding RNA transcriptome profile changes associated with SAPHO syndrome and inspiration for further investigation on clinical biomarkers and molecular regulators of this inadequately understood clinical entity.

## Introduction

Synovitis, acne, pustulosis, hyperostosis, and osteitis (SAPHO) syndrome is known as a rare inflammatory disease named by French rheumatologists in 1978 (Bjorksten et al., 1978), with an estimated prevalence of less than one in 10,000 (Magrey and Khan, 2009). The manifestations mainly include skin lesions as well as osteoarticular damages on anterior chest wall, the spine and pelvic bones, etc. Due to the lack of validated diagnostic criteria, the diagnosis and treatment for SAPHO syndrome has been controversial (Furer et al., 2020) and mainly based on clinical and radiological findings (Jurik et al., 2018). The similarity to other autoimmune diseases and cooccurrence of manifestations of other immune-mediated diseases indicated that disturbance of autoimmune system may contribute to its etiology (Naves et al., 2013; Marzano et al., 2016).

Neutrophils have been observed to play important roles in SAPHO syndrome. Enhanced neutrophil infiltration was observed in PSTPIP2-deficient mice which presented a SAPHO syndrome-like phenotype (Liao et al., 2015), while neutrophils purified from SAPHO patients were reported to have an abnormal internal oxidant production rather than intercellular oxidant production (Liu et al., 2016). Our previous study (Sun et al., 2019) focused on the differentially expressed genes in peripheral neutrophils from patients with SAPHO syndrome, indicating an over-active neutrophil recruitment in patients and possibly suggesting molecular candidates for further study on diagnosis and pathology of this disease. Emerging evidences indicated that long noncoding RNAs (lncRNAs) dysregulation may play important roles in autoimmune diseases such as psoriasis, rheumatoid arthritis, and Crohn’s disease (Wu et al., 2015), while lncRNA expression profiles in neutrophils revealed potential biomarker for renal involvement in SLE patients (Cai et al., 2021), providing new clues for functional biomolecules in SAPHO syndrome. Moreover, network analysis of lncRNAs and messenger RNAs (mRNAs) provided novel insights into the mechanism of competing endogenous RNA in immunomodulation (Feng et al., 2018). In this study, we performed whole-transcriptome sequencing and integrative bioinformatics analysis to identify key lncRNAs and mRNAs associated with SAPHO syndrome, hoping to find new functional molecules or biomarkers for this rare disease.

## Results

### Clinical Features of Included Individuals

Twelve patients with SAPHO syndrome [diagnosed according to the diagnostic criteria raised by Nguyen et al. (2012)] and 12 healthy volunteers were recruited between March 2017 and October 2019. The detailed clinical data of the 12 patients are shown in Table 1. The mean visual analog scale (VAS) of included patient was 5.33, indicating a middle-level pain of bones in patients. There was no significant difference in white blood cell or neutrophil count between SAPHO patients and healthy controls, while the peripheral levels of C-reactive protein (CRP) and ESR were higher than normal in patients (Table 2).

**TABLE 1:** Clinical data of the twelve SAPHO patients.

Patients	ESR (mm/h)	hsCRP (mg/L)	Osteocalcin (ng/L)	VAS (1–10)	White blood cell (*10^9^/L)	Neutrophil (*10^9^/L)
S1	2	0.17	1.54	2	6.34	3.98
S2	7	0.12	2.55	2	9.46	5.63
S3	16	0.12	3.5	5	9.75	5.49
S4	20	2.73	2.96	7	6.62	3.89
S5	21	7.2	5.43	8	7.04	5.01
S6	24	7.84	6.39	3	4.43	1.41
S7	14	4.01	3.06	8	2.67	1.15
S8	29	7.21	2.37	9	10.61	6.42
S9	72	26.15	1.88	9	6.52	4.25
S10	9	2.52	7.05	1	8.15	4.65
S11	20	1.14	4.95	4	4.80	2.46
S12	40	7.84	0.29	6	5.42	3.42

*ESR, erythrocyte sedimentation rate; hsCRP, high-sensitivity C-reactive protein; VAS, visual analog scale.*

**TABLE 2:** Features of included individuals.

	Control	SAPHO	Reference range	*p*-Value
Gender (female/male)	9/3	9/3		1
Age	31.67 ± 12.39	35.58 ± 11.64		0.178
White blood cell (*10^9^/L)	6.30 ± 1.25	6.82 ± 2.36	4.0–10.0	0.510
Neutrophil (*10^9^/L)	3.29 ± 0.81	3.98 ± 1.65	1.8–6.3	0.204
VAS (1–10)	–	5.33 ± 2.90	0–10	–
hsCRP (mg/L)	–	5.59 ± 7.18	0–3	–
ESR (mm/h)	–	22.83 ± 18.52	0–20	–
Osteocalcin (ng/L)	–	3.50 ± 2.05	1.8–8.4	–

*SAPHO, synovitis, acne, pustulosis, hyperostosis, and osteitis.*

### Overview of RNA-seq and Identification of lncRNAs

**FIGURE 1:**
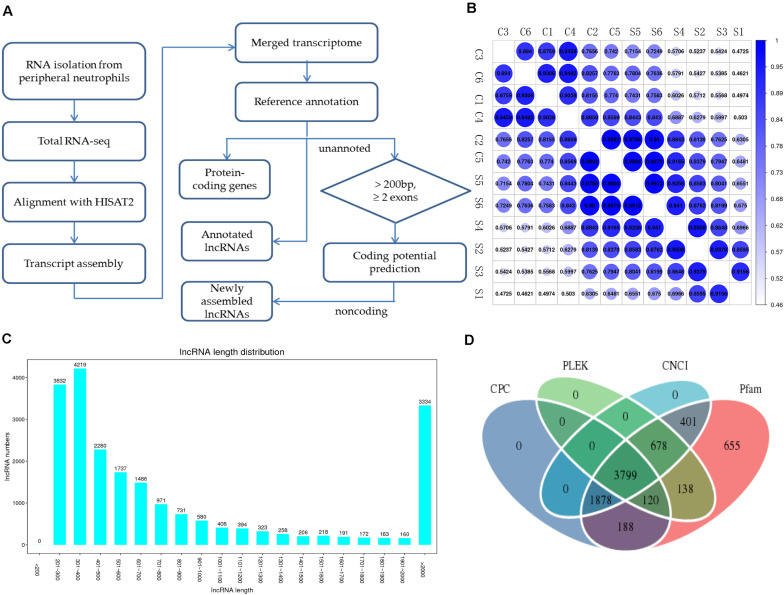
Overview of RNA-seq and lncRNA identification in patients with SAPHO syndrome. **(A)** Experimental and computational scheme for RNA-seq and identification of lncRNAs in the samples. **(B)** Correlation coefficient map between samples. **(C)** Average lncRNA length distribution of the 12 samples. **(D)** Venn diagram illustrating the distribution of the lncRNAs in the Pfam, CNCI, PLEK, and CPC software databases.

To systematically identify mRNAs and lncRNAs related to SAPHO syndrome, we selected 12 age- and sex-matched samples (six patients and six healthy controls) and conducted total RNA-seq. The detailed statistics of the sequencing data are shown in Table 3. Using lncRNAs in the Lncipedia database^1^ as a reference annotation, we assembled 21,663 candidate lncRNAs (including newly assembled lncRNAs) with a bioinformatics scheme (Figure 1A, see details in “Materials and Methods”), among which 3,700 were newly assembled lncRNAs. Sample correlation analysis of the expression of lncRNAs indicated a relatively closer relationship within group (Figure 1B). Potential noise in lncRNA identification was filtered according to genomic location and length (lncRNA length distribution is shown on Figure 1C), removing transcripts with coding potential based on calculation by four computational tools: pfam, CNCI, PLEK, and CPC (Figure 1D).

**TABLE 3:** Statistics of the sequencing data.

Sample	clean_reads	valid_bases	Q30	mapping_rate
C1	97.08 M	90.65%	95.43%	97.89%
C2	96.24 M	94.98%	96.16%	98.79%
C3	95.82 M	94.78%	96.14%	98.87%
C4	96.04 M	95.30%	96.11%	98.91%
C5	96.14 M	93.38%	95.82%	98.57%
C6	95.21 M	87.14%	95.15%	95.88%
S1	93.96 M	80.59%	94.13%	92.39%
S2	97.47 M	94.08%	96.36%	98.72%
S3	96.25 M	93.81%	95.73%	98.84%
S4	96.44 M	90.95%	95.92%	96.44%
S5	96.74 M	92.93%	96.15%	95.93%
S6	94.87 M	91.95%	94.92%	96.12%

### Differentially Expressed lncRNAs in Patients With SAPHO Syndrome

**FIGURE 2:**
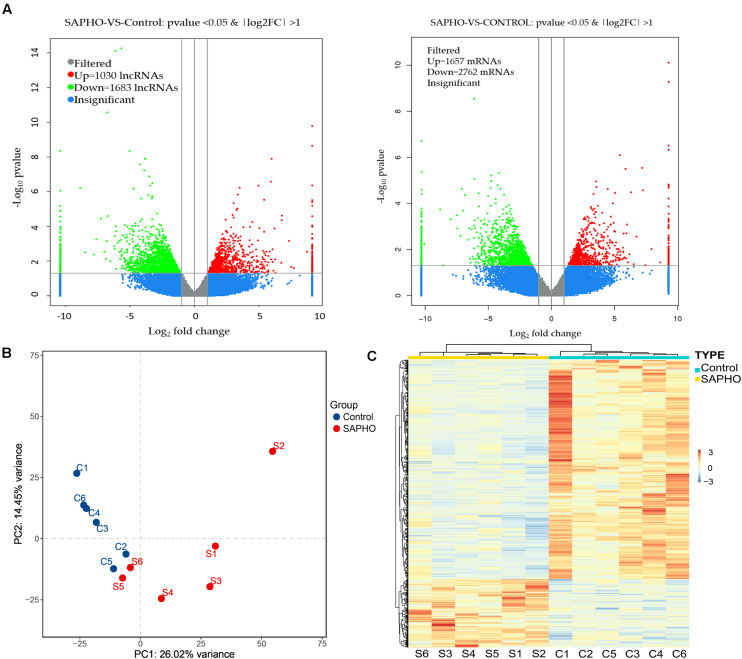
Differentially expressed lncRNAs and mRNAs in patients with SAPHO syndrome. **(A)** The differentially expressed lncRNAs (left) and mRNAs (right) were identified using a cutoff as “twofold expression difference and *p*-value < 0.05.” **(B)** PCA map of all samples in control and SAPHO group. **(C)** Hierarchical clustering analysis of DE lncRNA-targeted DE mRNAs in SAPHO patients showed closer relationship within both groups.

The differentially expressed mRNAs and lncRNAs were identified between SAPHO group and control group using a twofold expression difference and a *p-*value < 0.05 as a cutoff (Figure 2A). A total of 4,419 DE mRNAs (1,657 upregulated and 2,762 downregulated) were identified, while 2,713 lncRNAs (1,030 upregulated and 1,683 downregulated) were detected, among which clear distinction between samples in the two groups were revealed by principal component analysis (PCA) (Figure 2B). In addition, hierarchical clustering analysis was conducted to illustrate the distinguishable expression pattern of these DE lncRNA-targeted mRNAs between the two groups (Figure 2C). The top 60 DE lncRNA-targeted mRNAs are shown in Supplementary Table 1, including forkhead box O3 (FOXO3), signal-regulatory protein beta 1 (SIRPB1), interleukin 1 receptor type I (IL1R1), etc. FOXO3 has been reported to be important in the control of ROS levels in bone metabolic disorders *via* the activation of autophagy (Wang et al., 2020), while another member of the FOXO family, FOXO1, has been indicated in SAPHO syndrome (Berthelot et al., 2018).

### Coexpression Network of DE lncRNAs and mRNAs

**FIGURE 3:**
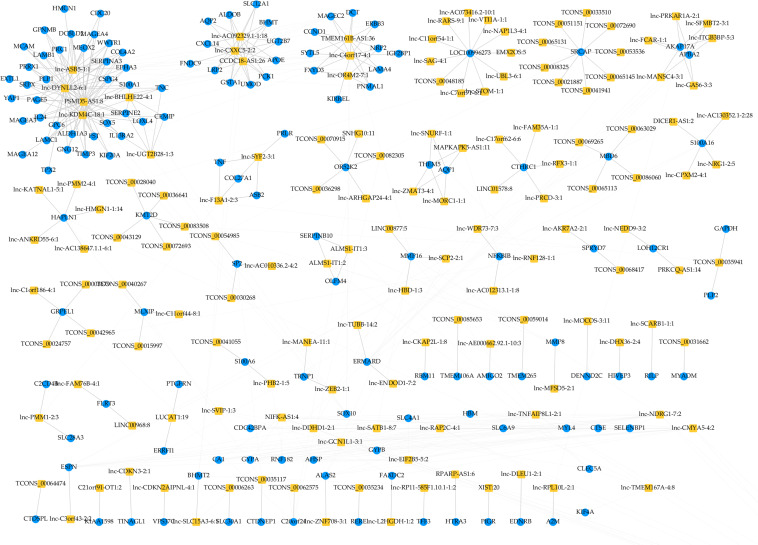
MCL clustering of coexpression lncRNA and mRNA.

To identify hub regulatory factors for SAPHO syndrome, the lncRNA-mRNA coexpression network was constructed based on Pearson’s correlation coefficients. Top 500 (according to *p*-value) connections are shown in Figure 3, which consisted of 281 nodes, including 139 differentially expressed mRNA and 142 differentially expressed lncRNAs. The 500 connections were further grouped into small clusters with Markov clustering (MCL) to detect highly interconnected lncRNA-mRNA networks (Figure 3). A total of 127 lncRNAs and 154 mRNAs were identified in clusters of the highly interconnected networks, and a GO analysis was conducted in these genes to predict the possible function of the lncRNA targets (Table 4). Interestingly, same as the GO analysis of differentially expressed mRNAs we previously reported (Sun et al., 2019), cell adhesion and cell migration were again significantly involved in that of the newly generated lncRNA target gene cluster, indicating that the migration of neutrophils may play important roles in the pathology of SAPHO syndrome.

**TABLE 4:** GO term enrichment of lncRNA target genes in clusters of the highly interconnected coexpression network.

Gene ontology term	Count	*p*-Value	Fold enrichment
Cell adhesion	12	0.0006	3.4842
Extracellular matrix organization	8	0.0007	5.4396
Cellular response to retinoic acid	5	0.0018	9.5193
Wound healing	5	0.0030	8.3294
Oxygen transport	3	0.0054	26.6540
Cell migration	6	0.0093	4.6489
Negative regulation of protein phosphorylation	4	0.0106	8.7390
Peripheral nervous system development	3	0.0136	16.6587
Amino-acid betaine metabolic process	2	0.0148	133.2698

### Inference of Potential Functions of DE lncRNAs

**FIGURE 4:**
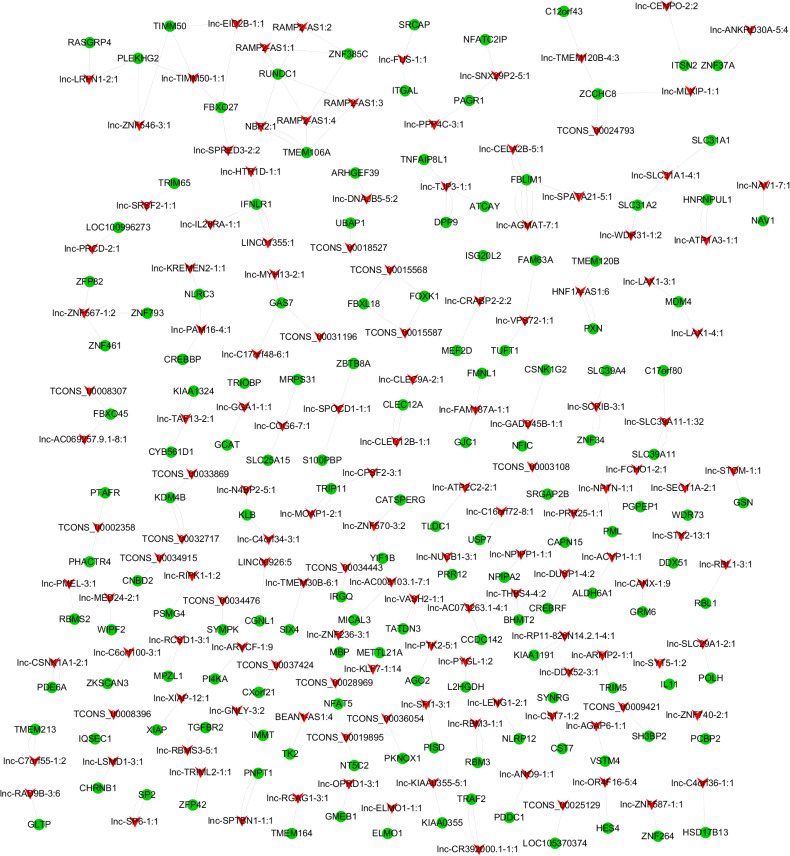
lncRNA-mRNA interaction network in a *cis* manner.

**FIGURE 5:**
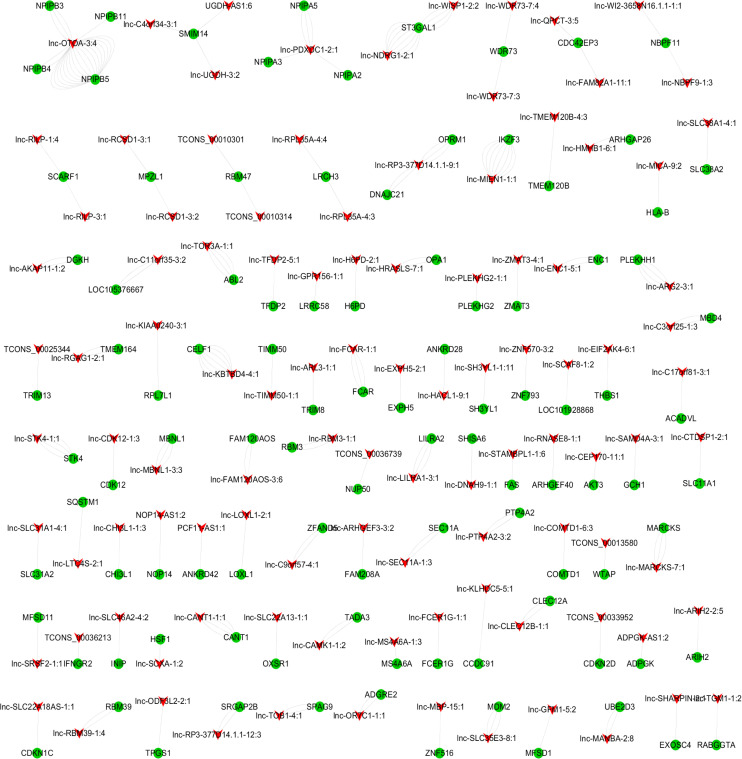
lncRNA-mRNA interaction network in a *trans* manner. Kyoto Encyclopedia of Genes and Genomes (KEGG) analysis indicated that the significantly (*p* < 0.05) enriched pathways in the lncRNA-targeted genes mainly included adipocytokine signaling pathway, ErbB signaling pathway, FoxO signaling pathway, etc. (Figure 6).

To predict the biological function of DE lncRNAs, we further conducted advanced analysis of lncRNA and mRNA interaction. As a result, 1,314 pairs of interacting lncRNA-mRNA in a *cis* manner as well as 9,351 pairs in a *trans* manner were found. The top 200 lncRNA-mRNA interaction pairs in *cis* and *trans* manner are shown in Figures 4, 5.

**FIGURE 6:**
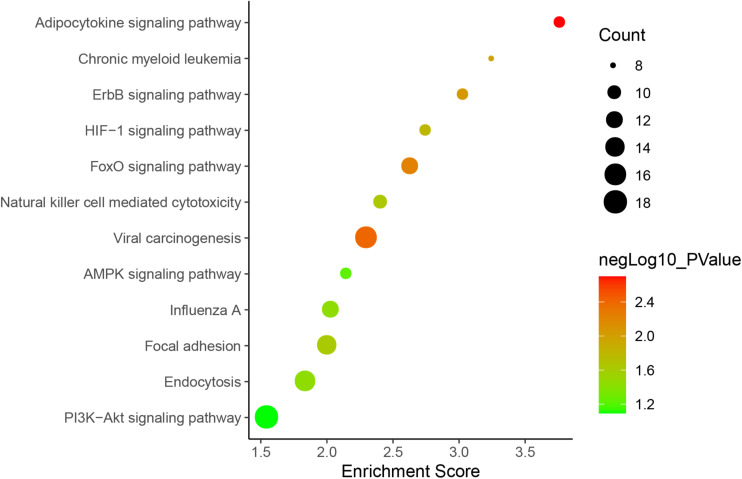
KEGG pathways involved in lncRNA-mRNA interaction network in SAPHO syndrome. A higher enrichment score and a lower *p*-value indicate stronger pathway enrichments. The top 10 significantly enriched terms of biological process, cellular component and molecular function in GO analysis are shown in Figure 7. Specifically, genes in the lncRNA-mRNA interaction network were enriched for terms related to production and function of miRNAs, indicating that DE lncRNAs may also function by regulating miRNAs to influence the expression of targeted mRNAs.

**FIGURE 7:**
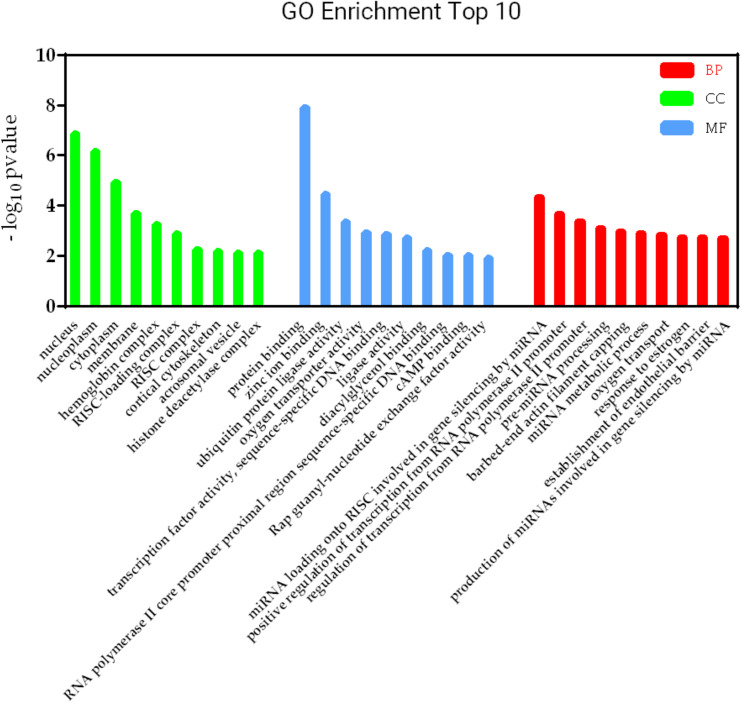
GO terms involved in lncRNA-mRNA interaction network in SAPHO syndrome. The top 10 significant GO terms in biological process are shown in red, while those in cellular component in green and molecular function in blue.

### Clinical Correlation Analysis of Coexpressed lncRNA-mRNA

**FIGURE 8:**
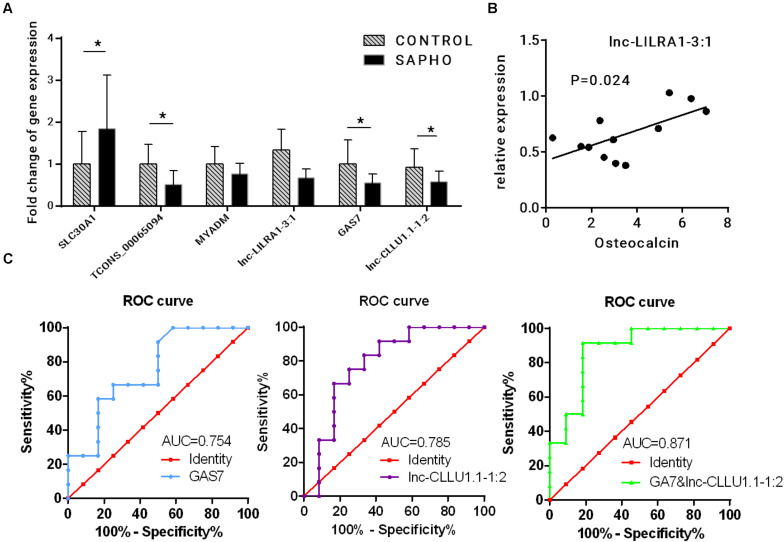
Verification of deregulated lncRNA and mRNA. **(A)** Relative expression of three coexpressed lncRNA-mRNA pairs by qRT-PCR in 12 patients and 12 healthy controls. **(B)** The correlation between the relative expression of lnc-LILRA1-3:1 and serum osteocalcin. **(C)** Analysis of predictive ability of differentially expressed RNAs for diseases.

Three coexpressed lncRNA-mRNA pairs were randomly selected for validation of the RNA-seq results by quantitative real-time PCR (qRT-PCR) in all 12 SAPHO patients and 12 healthy controls (Figure 8A). The expressions of SLC30A1 were upregulated while those of TCONS_00065094, GAS7, and lnc-CLLU1.1-1:2 were downregulated in qRT-PCR, which were in accordance with the RNA-seq data. Although the expression of lnc-LILRA1-3:1 was not significantly downregulated in SAPHO group as indicated in the RNA-seq data, it was positively related to serum Osteocalcin in the patients, providing a potential biomarker for disease progression (Figure 8B). To evaluate the diagnostic power of differentially expressed lncRNA and mRNAs identified in our study, the ROC curves of GAS7 and corresponding lnc-CLLU1.1-1:2 were generated to detect the diagnostic accuracy (Figure 8C). GAS7 and lnc-CLLU1.1-1:2 exhibited an AUC value of 0.754 (*p* < 0.01) and 0.785 (*p* < 0.01), respectively, in distinguishing SAPHO patients (*n* = 12) from healthy controls (*n* = 12), while the combined ROC curve of GAS7 and lnc-CLLU1.1-1:2 showed more reliable diagnostic ability with an AUC value of 0.871.

## Discussion

Our study focused on the deregulated mRNAs and lncRNAs in peripheral neutrophils from patients with SAPHO syndrome, trying to describe how the most abundant leucocytes, neutrophils, participate in SAPHO syndrome.

Construction of the lncRNA-mRNA coexpression network showed the potential regulating factors for SAPHO syndrome. We selected lncRNA target genes from the most highly interconnected lncRNA-mRNA cluster to study the function of deregulated lncRNAs. A total of 127 lncRNAs and 154 mRNAs were identified in the clusters of the highly interconnected networks and enriched in cell adhesion and cell migration, similar to the GO analysis results of differentially expressed coding genes (Sun et al., 2019).

Furthermore, the lncRNA-mRNA interaction networks for both *cis* and *trans* regulators were also constructed, with GO and KEGG analyses performed to gain an insight into the biological function of DE lncRNAs and their targets. Nine genes were enriched in the adipocytokine signaling pathway, while the roles of adipocytokines as diagnostic biomarkers in rheumatic disease has been suggested (Neumann et al., 2021), which showed strong modulatory properties on different effector cells in rheumatic joint diseases such as OA and RA (Carrión et al., 2019). Consistent with previous reports indicating FoxO1 in SPAHO syndrome (Berthelot et al., 2018), another nine genes including FoxO3 in the FoxO signaling pathway was also found to be regulated by DE lncRNAs in the SAPHO group. GO analysis showed that genes in the lncRNA-mRNA interaction network were enriched for terms related to production and function of miRNAs, which play important role in bone destruction of rheumatoid arthritis (Zhao et al., 2020), indicating that DE lncRNAs may also function by regulating miRNAs to influence the expression of targeted mRNAs.

Three coexpressed lncRNA-mRNA pairs were selected for qRT-PCR validation. The results of qRT-PCR were approximately in consistence with RNA-seq data. The ROC curve of GAS7 and Lnc-CLLU1.1-1:2 indicated that such pair may be a promising biomarker of SAPHO syndrome. However, the mechanism how GAS7 and Lnc-CLLU1.1-1:2 participate in the etiology and development of SAPHO syndrome requires further investigation.

## Conclusion

Our research performed integrated analysis of lncRNA-mRNA network to systematically screen potential biomarkers for SAPHO syndrome. Although with a limited number of cases, our study may help in providing novel diagnostic biomarkers as well as potential regulators in this rare clinical entity, which is characterized by diverse clinical manifestations. However, further efforts are still requested to address the role of these deregulated genes and pathways in SAPHO syndrome.

## Materials and Methods

### Subjects and Samples

This study was approved by the Institutional Review Board (IRB) of Peking Union Medical College Hospital (PUMCH). A total of 12 patients with SAPHO syndrome and 12 healthy volunteers in the same age range were recruited between March 2017 and October 2018 with informed consent obtained. Whole transcriptome sequencing was performed in six age- and sex-matched patients and healthy controls to detect differentially expressed mRNAs and lncRNAs. Twenty-milliliter serum blood sample was taken from each participant and neutrophils were isolated with polymorphprep by gradient centrifugation.

### Sequencing and Analysis of RNA Expression

The cDNA library was constructed by PCR amplification after qualified RNA (rRNA removed) processed to reverse transcription. RNA sequencing was conducted using Illumina HiSeq 2000 Platform. The original data were pretreated with Trimmomatic software (Bolger et al., 2014), with the reads in the whole process of quality control statistically summarized. Clean data was mapped to GRCh38 with HISAT2 (Kim et al., 2015) and then assembled into transcripts by StringTie software (Pertea et al., 2015). The lncRNAs in the assembled transcripts were identified using pfam_scan.pl^2^, CNCI^3^, PLEK (Li et al., 2014), and CPC v0.9-r2^4^. The expression level of RNAs was calculated by fragments per kilobase of exon per million fragments mapped (FPKM). Differentially expressed mRNAs and lncRNAs between the control and SAPHO groups were determined by DEseq with “fold change ≥ 2.00 and *p-*value < 0.05” as the threshold for significance.

### Quantitative Real-Time PCR

Relative expression of selected mRNAs and lncRNAs was determined by qRT-PCR. Total RNA was reverse transcribed to cDNA using a qPCR RT Master Mix with gDNA Remover Kit (TOYOBO). qRT-PCR was performed on Biorad iQ5 machine using a SYBR Green Realtime PCR Master MixPremix (TOYOBO) according to the manufacturer. All experiments were conducted in three technical replicates. The relative expression level of each target gene was determined by normalizing to the expression of β-actin with the 2^–ΔΔCt^ method. Primers are shown in Supplementary Table 2.

### GO Terms and KEGG Pathway Analysis

Targeted mRNAs of deregulated lncRNAs were selected for functional annotation. Functional pathway analysis was based on KEGG database^5^. Terms of biological process, cellular component, and molecular function were analyzed according to GO^6^ database, using hypergeometric distribution test to determine the significance of mRNA enrichment in each GO entry. The enrichment score was evaluated as follows:

$$\mathrm{Enrichment score}=\frac{m}{n}/\frac{M}{n}$$

Here, *N* is the number of GO- or KEGG-annotated mRNAs in all genes, while *n* is the number of GO- or KEGG-annotated mRNAs in DE lncRNA-targeted genes in *N*. *M* is the number of mRNAs annotated to a particular GO term or KEGG pathway in all genes while *m* is the number of genes annotated to a particular GO term or KEGG pathway in DE lncRNA-targeted genes.

### Coexpression Network Construction of lncRNA and mRNA

To illustrate the coexpression relationship of deregulated lncRNAs and mRNAs between groups, Pearson’s correlation coefficient was calculated (coefficients of 0.8 or greater are considered significant). We selected top 500 lncRNA-mRNA pairs (*p* < 0.05) to construct the coexpression network with the Cytoscape software. MCL was used to detect highly interconnected mRNA/lncRNA clusters.

### Cis and Trans Regulation Prediction

The DE lncRNAs were subjected to both *cis* and *trans* prediction. A *cis-*regulatory lncRNA exerts its function on a neighboring gene located at the same chromosome, while a *trans-*regulatory lncRNA does not meet the criterion. For *cis-*regulator prediction, we identified genomic localization of the paired lncRNA-mRNA and genes within 300 kb upstream or downstream the lncRNA which coexpressed either in the same direction of the lncRNA or not were regarded as the potential targets regulated by the lncRNA in a *cis* manner. For *trans-*regulator prediction, RNA interaction software RIsearch-2.0 (Alkan et al., 2017) was used to predict the binding of candidate lncRNA and mRNA at the nucleic acid level. The screening condition that the base number of direct interaction between two nucleic acid molecules is no less than 10, and the base binding free energy is no more than 50 is used to identify the directly interaction between lncRNA and mRNA. The prediction for *trans-*regulatory lncRNAs was based on the coexpression relationship (coefficients > 0.8 and *p* < 0.05) as well as the binding free energy between mRNA and lncRNA.

### Statistical Analysis

Continuous variables were presented as means ± standard deviation. Student’s *t*-test was performed using SPSS version 20.0 software for comparisons between groups. *p*-Value < 0.05 was considered statistically significant.

## Data Availability Statement

Original data of RNA-Seq has been submitted to www.ncbi.nlm.nih.gov/sra/(PRJNA517563). Data and materials are available from corresponding authors on reasonable request.

## Ethics Statement

The studies involving human participants were reviewed and approved by the Institutional Review Board of Peking Union Medical College Hospital. The patients/participants provided their written informed consent to participate in this study.

## Author Contributions

GH and TW: conceptualization and methodology. MZ, QL, and HJ: data curation. YS and CL: formal analysis. GH, YS, and CL: funding acquisition. YS: investigation and writing original draft. All authors contributed to the article and approved the submitted version.

## Conflict of Interest

The authors declare that the research was conducted in the absence of any commercial or financial relationships that could be construed as a potential conflict of interest.

## Footnotes

1 https://lncipedia.org/

2 http://pfam.xfam.org/

3 https://github.com/www-bioinfo-org/CNCI

4 http://CPC.cbi.pku.edu.cn

5 http://www.kegg.jp/

6 http://amigo.geneontology.org/amigo/landing
