# Supplementary material for: Integrative Analysis of lncRNA-mRNA Profile Reveals Potential Predictors for SAPHO Syndrome
Source: Front Genet. 2021 Jun 21;12:684520. doi: 10.3389/fgene.2021.684520 (PMC8255928; doi:10.3389/fgene.2021.684520)
Supplement: Supplementary file 1 [file Table_1.DOCX]

| *Supplementary talble 1* TOP 60 targeted mRNAs | | | | |
| --- | --- | --- | --- | --- |
| id | foldChange | pval | up_down | gene |
| XM_005260644.2 | 97.5770 | 2.70736E-11 | Up | SIRPB1 |
| XM_011545151.1 | 29.9828 | 2.42365E-10 | Up | ACER3 |
| NM_001110219.2 | 60.8654 | 2.5853E-10 | Up | GJB6 |
| XM_011515135.1 | 15.0139 | 8.43644E-10 | Up | ABCA13 |
| XM_005260641.2 | 14.5820 | 1.89629E-09 | Up | SIRPB1 |
| XM_006715511.2 | 0.0560 | 2.29173E-09 | Down | LMBRD1 |
| XM_011529130.1 | 19.8465 | 3.33808E-08 | Up | SIRPB1 |
| NM_001135844.2 | 500.1895 | 4.42035E-08 | Up | SIRPB1 |
| XM_011529131.1 | 94.5002 | 9.81187E-08 | Up | SIRPB1 |
| NM_001284254.1 | 7.0756 | 1.80121E-07 | Up | GUCD1 |
| XM_011530866.1 | 41.9576 | 2.33504E-07 | Up | UPRT |
| XM_005245323.1 | 0.0462 | 2.3785E-07 | Down | FAM63A |
| XM_005273426.2 | 0.0459 | 2.6945E-07 | Down | EGR3 |
| NM_001277818.1 | 0.0621 | 5.0518E-07 | Down | YTHDF3 |
| XM_011548261.1 | 0.0000 | 5.30114E-07 | Down | VPS52 |
| XM_011529136.1 | 0.0922 | 5.34005E-07 | Down | SIRPB1 |
| XM_006722927.1 | 9.3072 | 8.21647E-07 | Up | ATG4D |
| XM_011536287.1 | 0.0473 | 8.7992E-07 | Down | LOC101928868 |
| XM_005258192.3 | 9.0340 | 1.34236E-06 | Up | POLI |
| NM_001199382.1 | 0.1337 | 1.64859E-06 | Down | RNF145 |
| XM_011542062.1 | 0.1648 | 1.80177E-06 | Down | TNFRSF1B |
| NM_001184801.1 | 0.1790 | 2.21274E-06 | Down | UBR2 |
| XM_006714697.2 | 13.7065 | 2.21304E-06 | Up | CAST |
| XM_011521515.1 | 0.1806 | 2.43089E-06 | Down | IGF1R |
| XM_011520207.1 | 0.1006 | 2.58388E-06 | Down | TCP11L1 |
| NM_001185010.2 | 9.5932 | 2.59822E-06 | Up | RNF4 |
| NM_001300928.1 | 15.8591 | 2.74079E-06 | Up | SNX3 |
| XM_005255278.2 | 0.1033 | 2.81695E-06 | Down | UBN1 |
| XM_011524244.1 | 15.3887 | 3.16354E-06 | Up | SYNRG |
| XM_006719717.1 | 6.4569 | 3.56175E-06 | Up | RNF10 |
| XM_005250208.2 | Inf | 4.0141E-06 | Up | FBXL13 |
| XM_011545306.1 | 0.0615 | 4.30355E-06 | Down | PPFIA1 |
| XM_005264019.3 | 9.3576 | 4.40909E-06 | Up | C2orf49 |
| NM_004712.4 | 5.1345 | 7.59382E-06 | Up | HGS |
| XM_011519305.1 | 0.1557 | 8.09639E-06 | Down | NUDT5 |
| XM_011515381.1 | 0.1387 | 9.04872E-06 | Down | GPR141 |
| XM_011521514.1 | 0.2064 | 9.32171E-06 | Down | IGF1R |
| XM_011524258.1 | Inf | 9.61215E-06 | Up | TMC6 |
| XM_005270693.2 | 0.0000 | 9.74509E-06 | Down | SLC35A3 |
| XM_011544408.1 | 0.0370 | 9.98653E-06 | Down | LETM2 |
| XM_011527823.1 | 0.2050 | 1.10061E-05 | Down | MAST3 |
| XM_011539984.1 | Inf | 1.15676E-05 | Up | STAMBPL1 |
| XM_011535627.1 | 0.1413 | 1.16731E-05 | Down | FOXO3 |
| XM_005247462.3 | 5.0801 | 1.19869E-05 | Up | MBNL1 |
| NM_001163377.1 | 0.0408 | 1.23546E-05 | Down | QPCTL |
| XM_005276763.3 | 5.9939 | 1.28183E-05 | Up | CCDC125 |
| XM_011524245.1 | 0.0211 | 1.30852E-05 | Down | SYNRG |
| XM_011526294.1 | 0.0000 | 1.32794E-05 | Down | LOC105376875 |
| XM_011512823.1 | 0.1688 | 1.33878E-05 | Down | LPP |
| XM_011538497.1 | 0.1417 | 1.36407E-05 | Down | SSH1 |
| NM_001282981.1 | 0.1424 | 1.44541E-05 | Down | TLE3 |
| NM_000362.4 | 184.7026 | 1.50054E-05 | Up | TIMP3 |
| XM_011540549.1 | 6.5026 | 1.52739E-05 | Up | PADI2 |
| XM_005259825.2 | 0.2095 | 1.63765E-05 | Down | MAST3 |
| XM_005263930.3 | 53.2767 | 1.65741E-05 | Up | IL1R1 |
| NM_018212.4 | 0.0697 | 1.78466E-05 | Down | ENAH |
| NM_001287430.1 | 167.7251 | 1.80584E-05 | Up | UPP1 |
| XM_011517116.1 | 0.1021 | 1.84423E-05 | Down | FAM49B |
| XM_006716445.2 | 0.0441 | 1.85869E-05 | Down | SPIDR |
| XM_011527394.1 | 0.0091 | 1.88853E-05 | Down | ZNF528 |
