# Supplementary material for: Integrative Analysis of lncRNA-mRNA Profile Reveals Potential Predictors for SAPHO Syndrome
Source: Front Genet. 2021 Jun 21;12:684520. doi: 10.3389/fgene.2021.684520 (PMC8255928; doi:10.3389/fgene.2021.684520)
Supplement: Supplementary file 2 [file Table_2.DOCX]

| *Supplementary Table 2* Primers of differentially expressed RNAs | |
| --- | --- |
| NAME | SEQUENCE |
| SLC30A1-F | GGACAACTTAACATGCGTGGA |
| SLC30A1-R | ACACAAAAATCCCCTTCAGAACA |
| TCONS_00065094-F | CTGGACTGCTCACCTCCTCTT |
| TCONS_00065094-R | CTGGTTGCCTGATCTCCTGTA |
| MYADM-F | CCCTGTCTTGGCGCAACTT |
| MYADM-R | GGAACTGGACATAGGTGGTGG |
| lnc-LILRA1-3:1-F | CTACAAGATGCAGCCGGGAGG |
| lnc-LILRA1-3:1-R | AATGACCAGCATACAGTCCAGATA |
| GAS7-F | ATCTTTCCCATTGACCACTTTG |
| GAS7-R | CATCGGTTTCTAGCACGTTGA |
| lnc-LILRA1-3:1-F | CCCTTTTCGCAGTCACCTCT |
| lnc-LILRA1-3:1-R | TTGGGGCAAATTTCCAGAGC |
